# Using Task-fMRI to Explore the Relationship Between Lifetime Cannabis Use and Cognitive Control in Individuals With First-Episode Schizophrenia

**DOI:** 10.1093/schizbullopen/sgae016

**Published:** 2024-07-24

**Authors:** Tyler A Lesh, Joshua Rhilinger, Rylee Brower, Alex M Mawla, J Daniel Ragland, Tara A Niendam, Cameron S Carter

**Affiliations:** Department of Psychiatry and Behavioral Sciences, University of California, Davis, CA, USA; Department of Psychiatry and Behavioral Sciences, University of California, Davis, CA, USA; Department of Psychology, University of Minnesota, Minneapolis, MN, USA; Department of Psychiatry and Behavioral Sciences, University of California, Davis, CA, USA; Department of Psychiatry and Behavioral Sciences, University of California, Davis, CA, USA; Department of Psychiatry and Behavioral Sciences, University of California, Davis, CA, USA; Department of Psychiatry and Human Behavior, University of California, Irvine, CA, USA

**Keywords:** psychosis, neuroimaging, cognition, Marijuana

## Abstract

While continued cannabis use and misuse in individuals with schizophrenia is associated with a variety of negative outcomes, individuals with a history of use tend to show higher cognitive performance compared to non-users. While this is replicated in the literature, few studies have used task-based functional magnetic resonance imaging (fMRI) to evaluate whether the brain networks underpinning these cognitive features are similarly impacted. Forty-eight first-episode individuals with schizophrenia (FES) with a history of cannabis use (FES + CAN), 28 FES individuals with no history of cannabis use (FES-CAN), and 59 controls (CON) performed the AX-Continuous Performance Task during fMRI. FES+CAN showed higher cognitive control performance (dʹ-context) compared to FES-CAN (*P* < .05, η_p_^2^ = 0.053), and both FES+CAN (*P* < .05, η_p_^2^ = 0.049) and FES-CAN (*P* < .001, η_p_^2^ = 0.216) showed lower performance compared to CON. FES+CAN (*P* < .05, η_p_^2^ = 0.055) and CON (*P* < 0.05, η_p_^2^ = 0.058) showed higher dorsolateral prefrontal cortex (DLPFC) activation during the task compared to FES-CAN, while FES+CAN and CON were not significantly different. Within the FES+CAN group, the younger age of initiation of cannabis use was associated with lower IQ and lower global functioning. More frequent use was also associated with higher reality distortion symptoms at the time of the scan. These data are consistent with previous literature suggesting that individuals with schizophrenia and a history of cannabis use have higher cognitive control performance. For the first time, we also reveal that FES+CAN have higher DLPFC brain activity during cognitive control compared to FES-CAN. Several possible explanations for these findings are discussed.

## Introduction

Cannabis is highly prevalent among individuals with schizophrenia, with studies suggesting 42% of patients use in their lifetime^1^ and 26% meet the criteria for a cannabis use disorder.^2^ Furthermore, the use of cannabis is approximately twice the rate of the general population.^3^ The relationship between cannabis use and psychosis has been receiving increasing attention as cannabis availability and use has been increasing in many parts of the world. This investigation is important given evidence that cannabis use, particularly in childhood or early adolescence, is associated with an increased risk of developing psychosis.^4^ Individuals at clinical high risk (CHR) for developing a psychotic disorder who also use cannabis have been shown to have an increased likelihood to transition to psychosis when use starts earlier and is more frequent^5^ although several studies have failed to show that cannabis use vs nonuse increases transition to psychosis.^5–7^ Cannabis use has also been associated with increased incidence of psychotic-like experiences (PLEs) in adolescents^8^ and a twin study by Karcher and colleagues^9^ suggests that the association between cannabis use and PLEs has a significant shared genetic component. Recent work by Di Forti and colleagues^10,11^ found that using cannabis with high levels of delta-9-tetrahydrocannabinol (THC) and patterns of higher use was associated with psychosis risk in a dose-dependent fashion. While this association is not well understood, there is some evidence of genetic overlap in risk for cannabis use and schizophrenia^12,13^ as well as of alterations in the endocannabinoid system in individuals with schizophrenia.^14^ Such evidence might highlight biological factors that underlie the relationship between cannabis use and psychosis risk.

In addition to the suspected role of cannabis in contributing to the risk of psychotic disorders, particularly in vulnerable individuals, cannabis use in individuals with schizophrenia is typically associated with poor outcomes. In particular, early in the course of illness, individuals who continue to use cannabis tend to show increased risk of, number of, and length of relapses.^15^ Continued cannabis use after the first episode has also been linked to worse positive symptoms and functioning compared to those who discontinued use.^16^ Early laboratory studies highlighted the effect of acute THC administration on exacerbating positive and negative symptoms of schizophrenia and also negatively impacting cognitive performance.^17^ In line with this, regular use of cannabis in healthy adults has generally been linked to lower cognition.^18–20^ However, a somewhat paradoxical but replicated finding in individuals with schizophrenia is that lifetime cannabis use has been linked to higher performance on executive functioning tasks, including attention, processing speed, and working memory.^21,22^ Several meta-analyses have reinforced this result in finding higher cognitive performance in lifetime cannabis users with schizophrenia.^23–25^

While studies of cognition using neuropsychological tests in cannabis using vs never using individuals with schizophrenia are prevalent, there are very few studies using functional neuroimaging to better understand the underlying functional brain circuitry in individuals with psychosis. One study in a large group of individuals at CHR found no evidence for thalamo-cortical resting-state connectivity differences in cannabis using and non-using CHRs.^26^ Sami and colleagues^27^ found evidence for hyperconnectivity in visual attention and visual-dorsal attention network interconnectivity in early psychosis individuals with cannabis use. In particular, while non-using individuals with psychosis showed a negative correlation between these networks and PANSS-positive symptoms, cannabis using individuals with early psychosis lacked this relationship. In contrast, Peeters and colleagues^28^ focused on DLPFC connectivity during resting state in individuals with non-affective psychosis, siblings, and controls, but found no differential influence of cannabis use on fronto-parietal network connectivity. In terms of task-based fMRI, studies of individuals with established schizophrenia and co-occurring cannabis abuse have shown increased cingulate and prefrontal activity during emotional memory^29^ and increased parietal activity during mental rotation.^30^ Other work has focused on reward-based performance and functional activation and shown higher reward-based behavioral sensitivity and greater thalamus and insula activity in non-using first-episode schizophrenia individuals compared to currently using individuals.^31^ One of the few task-fMRI studies comparing past schizophrenia cannabis users to non-users^32^ identified higher frontal and parietal brain activity in users during a dichotic listening task.

The present study seeks to use task-fMRI during cognitive control to further explore the relationship between cannabis use and cognition in individuals with schizophrenia within the first year of the first episode of psychosis. A sample of individuals with first-episode schizophrenia with a history of cannabis use (FES + CAN), but no current use or history of other substance use disorders, was compared to a group of never using first-episode schizophrenia patients (FES-CAN) and never-using controls (CON). Primary aims focused on comparing the 2 patient groups and exploratory analyses included comparisons with CON as well as examination of relationships between cannabis use metrics and clinical features. Based on the existing literature, we hypothesized that FES+CAN would show higher performance on a measure of cognitive control (dʹ-context) as well as greater prefrontal activation during the AX-Continuous Performance Task (AX-CPT) compared to FES-CAN. Additionally, CON who never used cannabis were hypothesized to show higher performance and greater prefrontal cortex activation compared to both patient groups.

## Methods

### Parent Study Design

Data analyzed in the present manuscript represent a subset of data collected between 2005 and 2013 as part of a larger study of cognition and FES (see supplementary material). Inclusion criteria were: (1) age 12–35, (2) schizophrenia, schizoaffective, or schizophreniform disorder, (3) onset of psychosis within 1 year. Matched controls were recruited from the community. All participants were assessed using the Structured Clinical Interview for the DSM-IV-TR.^33^ Exclusion criteria for all groups included: (1) Wechsler Abbreviated Scale of Intelligence (WASI) IQ score below 70, (2) positive urine toxicology screen for illicit drugs at the time of testing, (3) prior head trauma worse than a Grade I concussion, or (4) contraindication to MRI scanning. In the event of a positive toxicology result, participants were asked to refrain from using the substance for at least 3 weeks and return for an additional drug screen before the scan appointment. Individuals who failed multiple drug screens or met the criteria for current substance abuse or dependence were excluded. CONs were excluded for the following *additional* criteria: any lifetime diagnosis of an Axis I or Axis II disorder or any first-degree relatives with a psychotic disorder. After a complete description of the study to the subjects, written informed consent was obtained. The protocol was approved by the University of California, Davis Institutional Review Board (Study #226043), and all subjects were paid by check for their participation ($25/h for clinical appointments and $35/h for MRI appointments).

### Present Study Design

While the aims of the parent study above did not explicitly focus on cannabis use, data related to substance use were collected as part of study procedures. The subset of participants analyzed in the present dataset were selected from the larger dataset based on the following additional criteria: (1) presence of fMRI data, (2) reliable data regarding cannabis use history (eg, SCID-IV Module E, medical records, and participant report at the time of drug testing), and (3) absence of other substance abuse or dependence diagnosis. Individuals with any history of other (non-cannabis) substance use disorders were excluded in order to reduce confounds associated with other substance use. Given that all participants needed to test negative for all substances at the time of testing, individuals in the FES+CAN group had either already discontinued the use of cannabis or were able to discontinue use for several weeks to participate. Based on the above criteria, 48 FES+CAN (42 schizophrenia, 3 schizoaffective, and 3 schizophreniform), 28 FES-CAN (24 schizophrenia, 4 schizoaffective), and 59 CON without a history of cannabis use were identified (table 1).

**Table 1.** Demographic and Clinical Characteristics

	Group		
	FES-CAN (*N* = 28)	FES+CAN (*N* = 48)	CON (*N* = 59)
Age in years^a^	19.18 (4.20)	20.35 (2.58)	19.7 (3.47)
Gender (M/F)	16/12	45/3	30/29
Years of education	11.70 (2.79)	12.3 (1.68)	12.89 (2.61)
Years of parental education	14.04 (3.02)	14.54 (2.44)	14.55 (2.39)
WASI IQ	98.07 (12.46)	101.23 (14.03)	115.23 (10.72)
Age of psychosis onset	18.64 (4.25)	19.94 (2.47)	—
Duration of illness in years	0.53 (0.28)	0.53 (0.34)	—
Poverty	13.07 (5.76)	14.09 (5.03)	—
Disorganization	6.04 (2.29)	6.57 (3.23)	—
Reality distortion	14.50 (7.10)	16.87 (6.87)	—
Global assessment of functioning	45.79 (9.24)	45.23 (9.94)	—
Chlorpromazine equivalent (mg)	227.2 (210.9)	255.5 (173.3)	—
Current antipsychotic medication (Y/N)	23/5	38/10	—
Tobacco smoking (Y/N)	3/21	16/18	—
Age of first cannabis use^b^	—	15.49 (2.08)	—
Frequency of cannabis use^c^	—	30.15 (28.47)	—

*Note:* FES-CAN, lifetime cannabis never users with schizophrenia; FES + CAN, lifetime cannabis users with schizophrenia; CON, never using controls.

^a^Age range of 14–32.

^b^Comprises 39 participants with complete data.

^c^Comprises 27 participants with complete data; Number of uses per month during period of heaviest use.

Standard deviations are presented in parenthesis.

### Measures and Data Analysis

Clinical ratings were collected in the patient sample using the Scale for the Assessment of Negative Symptoms (SANS),^34^ Scale for the Assessment of Positive Symptoms (SAPS),^35^ and Brief Psychiatric Rating Scale (BPRS).^36^ These scales were used to compute Reality Distortion, Disorganization, and Poverty Syndrome Scales.^37^ Global functioning, which consists of a 0–100 score based on symptom presentation, role functioning, and social functioning, was assessed using the Global Assessment of Functioning Scale (GAF).^38^ Duration of illness was defined as the number of days between the first threshold psychotic symptom presentation and scan date, which was based on all available information (ie, parent/subject report, medical records). In addition to the binary coding of past cannabis use and never-use, the age of onset of cannabis use and peak monthly frequency of use was gathered for the majority of FES+CAN. Tobacco smoking status was also collected for a majority of the patient groups due to the potentially confounding effects of nicotine.

The AX-CPT has been described in detail previously,^39^ and the specific task parameters utilized in the present study have been described previously.^40^ Briefly, subjects are presented with a series of cues and probes and are instructed to make a target response (index finger button press) to the probe letter X only if it was preceded by the cue letter A. All cues and nontarget probes require nontarget responses (middle finger button press). Target sequence trials are frequent and set up a prepotent tendency to make a target response when the probe letter X occurs. As a result, nontarget sequence trials where any non-A cue (collectively called B-cues) is presented and followed by a probe letter X require the most cognitive control.

A specific measure of cognitive control performance, dʹ-context,^39^ was computed from AX hits and BX false alarms and group differences were tested using a univariate general linear model (GLM) covarying for age and sex. An additional GLM was conducted with tobacco use added as a covariate for the subsample of individuals for whom this data was available (approximately 75% of the patient sample; table 1). Demographic variables were tested using one-way ANOVA or Pearson chi-square, followed by post-hoc tests (least significant difference) when the null hypothesis was rejected. Clinical variables only in the patient groups were tested using independent samples *t*-tests or Pearson chi-square tests.

### Functional Imaging Parameters and Data Analysis

Imaging data were obtained using a 1.5T GE Signa system (see supplementary material). Preprocessing was completed using Statistical Parametric Mapping-12 (SPM12, http://www.fil.ion.ucl.ac.uk/SPM12), including slice timing correction, spatial realignment, spatial normalization to the Montreal Neurological Institute (MNI) EPI template using a rigid-body transformation followed by non-linear warping, and spatial smoothing using a Gaussian 8-mm full-width half-maximum kernel. Individual fMRI runs were removed from the analysis if scan-to-scan movement exceeded 0.45 mm based on average framewise displacement using the fsl_motion_outliers script. Functional imaging analysis was performed in SPM12 using the GLM. All trial types were modeled and only correct responses were included in the reported contrasts. Regressors included all cues, probes, and error trials. Translational and rotational movement data were included as covariates of noninterest. Group-level random-effects comparisons were performed between groups for the AX-CPT contrast subtracting the A cue from the B cue (CueB-CueA contrast) to measure activation under conditions of high vs low cognitive control. Contrasts were thresholded at the voxel level (*P* < .001) and clusters were considered significant if they survived FWE correction (*P* < .05). Left and right dorsolateral prefrontal cortex (DLPFC) regions of interest were prescribed a priori and obtained from the middle frontal gyrus labels of the Wake Forest University Pickatlas.^41^ Mean parameter estimates for the CueB-CueA contrast were extracted for each participant. Univariate GLMs with age and sex as nuisance covariates were used to test for group differences in left and right DLPFC. As with dʹ-context, a sub-analysis was conducted adding tobacco use as an additional covariate to ROI GLMs.

## Results

### Demographic and Sample Characteristics

The initial sample consisted of 32 FES-CAN, 53 FES+CAN, and 64 CON. However, after excluding participants due to excessive in-scanner movement (4 FES-CAN, 4 FES+CAN, 3 CON) or poor behavioral performance (1 FES+CAN, 2 CON), the final sample of 28 FES-CAN, 48 FES+CAN, and 59 CON remained (see table 1). One-way ANOVA revealed no significant group differences in age (*F*(2,135) = 1.163, *P* = .316), participant education (*F*(2,135) = 2.534, *P* = .083), or parental education (*F*(2,132) = 0.460, *P* = .632). Significant group differences were identified in WASI IQ (*F*(2,131) = 24.839, *P* < .001) with higher scores in CON compared to both FES+CAN (*P* < .001) and FES-CAN (*P* < .001). Pearson Chi-Square revealed a significant group difference in sex (*X*^2^ (2,135) = 23.87, *P* < .001) with a higher proportion of male participants in the FES+CAN compared to both FES-CAN (*P* < .001) and CON (*P* < .001). FES+CAN were also more likely to have a history of tobacco smoking compared to FES-CAN (*X*^2^ (1,58) = 7.63, *P* = .006). All other clinical comparisons between the 2 FES groups were not significant (*P* > .148).

### Behavioral Results

**Fig. 1.**
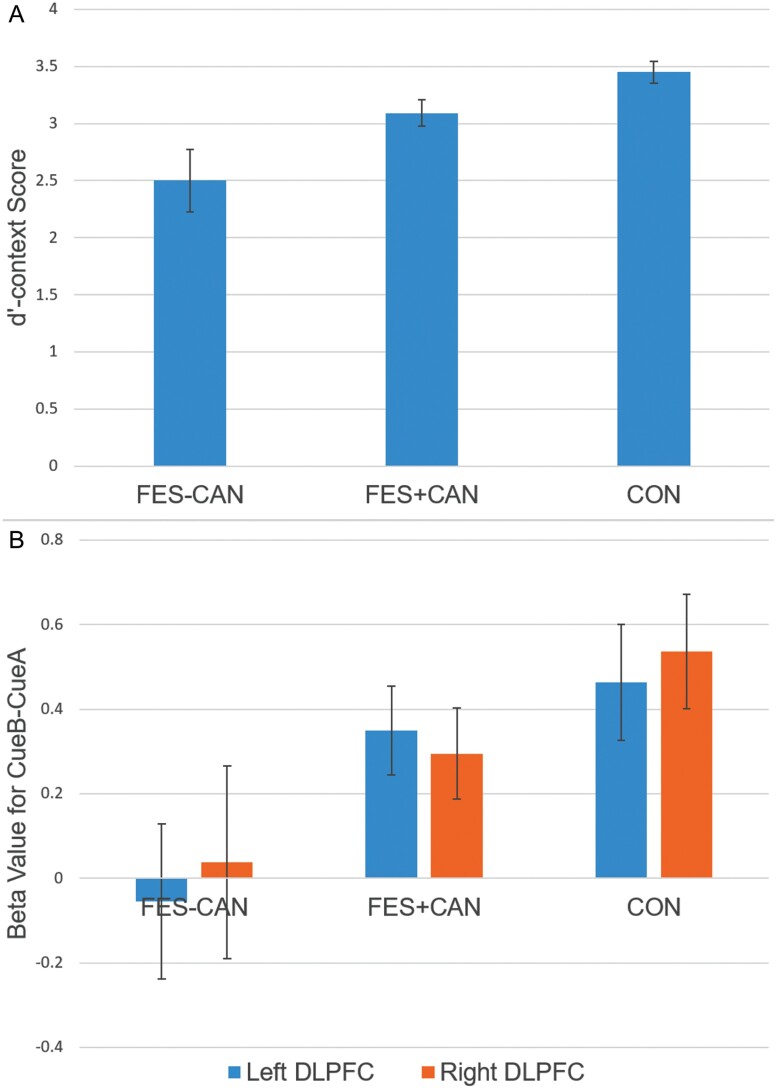
Top panel illustrates dʹ-context score across three groups. Bottom panel illustrates left and right DLPFC beta values for the CueB vs CueA contrast during the AX-CPT. FES-CAN, cannabis never users with schizophrenia; FES+CAN, lifetime cannabis users with schizophrenia; CON, control never users.

The primary hypothesis of interest was to test for differences in dʹ-context performance in FES+CAN vs FES-CAN. After covarying for age and sex, FES+CAN showed significantly higher performance on dʹ-context compared to FES-CAN (*F*(1,76)=4.012, *P* < .05). This finding remained significant after additionally controlling for tobacco smoking (*F*(1,58)=4.939, *P* < .05). Furthermore, CON showed significantly higher dʹ-context compared to both FES+CAN (*F*(1,107) = 5.270, *P* < .05) and FES-CAN (*F*(1,87) = 22.844, *P* < .001, figure 1).

### fMRI Results

Independent samples *t*-tests of movement metrics (mean framewise displacement) between groups revealed no significant differences between any group (group means: FES-CAN = 0.166, FES+CAN = 0.153, CON = 0.157; all *P* > .47). Analyses of left and right a priori DLPFC ROIs revealed significantly higher activity in FES+CAN vs FES-CAN in the left (*F*(1,76) = 4.183, *P* < .05) but not right (*F*(1,76) = 2.063, *P* = .155) hemisphere. These findings were unchanged after additionally controlling for tobacco smoking in the left (*F*(1,58) = 4.109, *P* < .05) and right (*F*(1,58) = 2.379, *P* = .129) DLPFC. CON showed significantly higher activity in both left (*F*(1,87) = 5.097, *P* < .05) and right (*F*(1,87) = 4.148, *P* < .05) DLPFC compared to FES-CAN. However, CON and FES+CAN DLPFC activity did not differ in the left (*F*(1,107) = 1.041, *P* = 0.310) or right hemisphere (*F*(1,107) = 2.525, *P* = .115, figure 1).

**Fig. 2.**
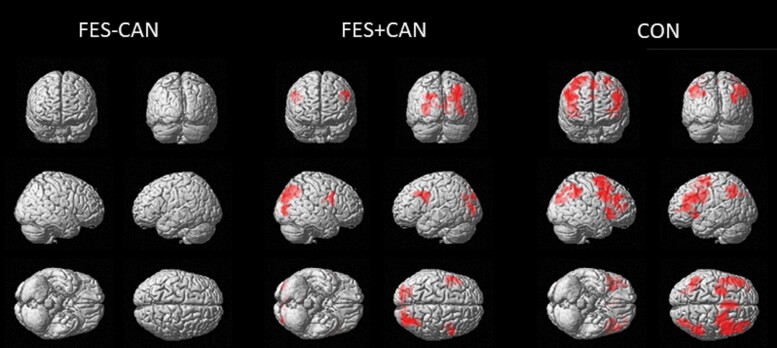
Within-group whole-brain maps of the CueB vs CueA contrast, significant clusters are displayed based upon a voxel level threshold of *P* < .001 and FWE cluster correction of *P* < .05. FES-CAN, cannabis never users with schizophrenia; FES+CAN, lifetime cannabis users with schizophrenia; CON, control never users.

For whole-brain voxelwise analyses (figure 2), CON participants showed robust activation of the fronto-parietal network with clusters in bilateral middle frontal gyri, left precentral gyrus, and bilateral superior parietal cortex reaching statistical significance. The FES+CAN group showed qualitatively less activation with significant clusters in the right superior parietal cortex, left superior occipital cortex, left middle frontal gyrus, and right precentral gyrus. No clusters survived correction in the FES-CAN group. Finally, no statistically significant clusters emerged between any group comparison at the whole-brain level (*P* < .001, *P* < .05 FWE cluster corrected). Significant clusters and their coordinates are presented in table 2.

**Table 2.** Significant Clusters That Survived a Voxelwise Statistical Threshold of *P* < .001 Followed by *P* < .05 FWE Cluster Correction

Group	Region	Cluster Size (mm^3^)	MNI Coordinates			T voxel peak
			x	y	z	
FES-CAN	No significant clusters					
FES+CAN	Right superior parietal	16 424	32	−78	34	6.22
	Left superior occipital	10 904	−20	−90	34	4.55
	Left middle frontal gyrus	5576	−50	18	38	4.43
	Right precentral gyrus	4640	42	6	26	4.36
CON	Right middle frontal gyrus	45 216	56	8	40	6.69
	Left middle frontal gyrus	22 256	−50	22	30	5.68
	Right supramarginal gyrus	11 392	52	−44	42	5.17
	Left superior parietal	8392	−28	−62	40	4.88
	Left precentral gyrus	5288	−26	−10	72	4.57
FES+CAN vs FES-CAN	No significant clusters					
CON vs FES+CAN	No significant clusters					
CON vs FES-CAN	No significant clusters					

*Note:* FES-CAN, lifetime cannabis never users with schizophrenia; FES + CAN, lifetime cannabis users with schizophrenia; CON, never using controls.

### Exploratory Correlations With Cannabis Use Metrics

Pearson bivariate correlations (Spearman’s rho for data violating normality assumptions) were used to explore the relationship between cannabis use metrics, such as the age of first cannabis use and frequency of use, with IQ, symptomatology (reality distortion, poverty, and disorganization), and functioning (GAF). Significant positive correlations were identified between the age of first cannabis use and both WASI IQ (*r* = 0.361, *P* < .05) and GAF (*r* = 0.364, *P* < .05). A significant positive correlation was also identified between the frequency of cannabis use and reality distortion symptoms (*r*_s_ = 0.430, *P* < .05) at the time of the scan.

## Discussion

As predicted, the present study found higher cognitive control performance on the AX-CPT task in FES+CAN compared to FES-CAN. CON showed higher cognitive control performance compared to both patient groups. FES+CAN also showed higher DLPFC activation on high vs low cognitive control trials compared to FES-CAN. Interestingly, while CON participants showed higher DLPFC activation compared to FES-CAN, there were no differences between CON and FES+CAN DLPFC activity. Higher cognition in past cannabis users with schizophrenia has been repeatedly found,^16,24,25,42–45^ although this may be the first fMRI study highlighting group differences on the AX-CPT.

In terms of task-based fMRI, there are very few studies that evaluate the impact of cannabis use in psychosis. Bourque and colleagues^29^ employed an emotional memory task and found higher cingulate and prefrontal activity a sample of 14 schizophrenia patients with co-occurring cannabis abuse compared to 14 schizophrenia non-users. The same samples also performed a mental rotation task and the schizophrenia cannabis-abusing group showed higher parietal activity compared to non-users.^30^ Loberg et al^32^ compared 13 past schizophrenia cannabis users to 13 non-users and identified higher frontal and parietal brain activity during a dichotic listening task, particularly during the active phases of the task. The present study is largely consistent with these findings, with higher DLPFC activity in individuals with schizophrenia and a history of cannabis use. In contrast, a recent study by Fish and colleagues^31^ used a combined variant of Monetary Incentive Delay and Eriksen flanker tasks and found higher reaction time reward sensitivity and greater activation in the thalamus and insula in schizophrenia non-users compared to current cannabis users with schizophrenia. However, this pattern of behavior and regional brain activity showed more similarity between cannabis-using individuals with schizophrenia and non-using control participants, which is partially consistent with the existing literature.

We also identified a significant relationship between the age of onset of cannabis use and both WASI IQ and GAF, such that earlier age of initiation was associated with lower current IQ and lower functioning. Earlier age of onset of cannabis use has been consistently associated with worse cognitive performance in a variety of domains including visual attention, inhibition, and verbal fluency.^46^ Buchy and colleagues^47^ found a similar relationship between age of cannabis initiation and IQ in cannabis-using CHRs. In a subset of our sample, we also found a significant relationship between the peak frequency of cannabis use and current symptomatology, which suggested that more frequent historical use was associated with more severe positive symptoms of psychosis. These findings are consistent with studies that have generally found more severe positive symptoms in cannabis users with psychotic disorders compared to non-users.^16,48–52^ In general, the present data suggest that although lifetime cannabis users showed higher cognition, within the cannabis using group itself, a more severe pattern of cannabis use was linked to largely negative features. These findings are in agreement with research that highlights the negative impact of continued cannabis use on outcomes in individuals with schizophrenia.^16,53^ Importantly, attempts to reduce cannabis use in first-episode psychosis patients have received increasing attention in recent years. Cognitive behavioral therapy with a specific focus on cannabis cessation and psychosis prevention has shown significant promise at not only reducing cannabis use and positive symptom severity, but also improving functioning compared to treatment as usual.^54^

Several theories have been proposed to explain why individuals with schizophrenia and a history of cannabis use display higher cognitive control than non-using individuals. Firstly, individuals with psychosis who used cannabis may have had higher social functioning and premorbid IQ prior to illness onset. Use is typically initiated in early to mid-adolescence; obtaining cannabis would presumably require strong social skills to identify a seller, and typical patterns of use involve social groups.^24,55,56^ Ferrero and colleagues^57^ found evidence of higher premorbid social functioning in both daily and occasional cannabis users with psychosis in a large European sample of first-episode individuals. This pattern of higher premorbid social functioning has also been seen in earlier studies,^58^ although some highlight higher premorbid IQ in past cannabis users with psychosis^42,57^ while others show no differences.^21^ Consistent with this theory, Leeson and colleagues^59^ found that the higher current IQ, verbal learning, and working memory in cannabis users vs non-users with first-episode schizophrenia were made nonsignificant after covarying for premorbid IQ.

Another theory that may explain the findings is the potential neuroprotective effects of cannabidiol (CBD), a major nonpsychoactive component of cannabis. CBD has anti-inflammatory effects and may have antipsychotic properties,^60^ which could be helpful in reducing inflammation that may be contributing to symptoms.^61^ CBD prescribed as a monotherapy^62^ or adjunctive has shown a modest effect in relieving positive symptoms in approximately half of clinical trials,^63^ although effects on cognition are typically not reported. While only trend-level, McGuire and colleagues^64^ did report some influence of adjunctive CBD treatment on cognitive performance (BACS) in addition to significant effects on positive symptoms and clinician ratings of improvement. Although the putatively beneficial effects of CBD could potentially inform the literature on cannabis and cognition in psychotic disorders, a significant challenge to this theory is that CBD levels in cannabis have been steadily declining over the past decades. CBD concentrations in cannabis over the last 20 years have shown a drop in the CBD:THC ratio to approximately 1:80 vs approximately 1:10 prior to 2005.^65^ To consume cannabis high in CBD, individuals would need to specifically seek out strains that have these properties, which is unlikely for individuals in the current study who were recruited largely prior to the availability of CBD-heavy strains (eg, Charlotte’s Web). Based on tests of seized cannabis during that time, we may speculate THC levels ranged from 8% to 14%.^66,67^

Lastly, an alternative hypothesis is that individuals who use cannabis and develop schizophrenia may have a lower vulnerability to psychosis compared to individuals who develop schizophrenia without any co-occurring use.^68^ Lower vulnerability might be reflected in more preserved cognition and neurobiology, which could be consistent with higher performance and brain activity in individuals with a history of cannabis use. Based on this perspective, individuals in this group might not have developed a psychotic disorder in the absence of cannabis use, although this is speculative. Studies conducted by the Bipolar-Schizophrenia Network on Intermediate Phenotypes (B-SNIP) may be considered consistent with this theory particularly with the discovery of Biotype 3 (B3). Individuals in B3 tend to be characterized by significant adolescent cannabis use^69^ in the context of relatively preserved cognition and function.^70^ Furthermore, B3 group membership has been associated with less genetic risk for schizophrenia based on lower psychosis polygenic risk scores.^71^ Other studies of polygenic risk scores for schizophrenia have highlighted that cannabis use in more genetically vulnerable individuals is associated with higher odds of more severe psychotic symptoms.^72^ Risk for schizophrenia and cannabis use^12,13^ or abuse^73,74^ has also been demonstrated to have significant genetic overlap. However, there is insufficient data to disentangle the independent or overlapping genetic signatures of cognitive factors, cannabis use, and risk for schizophrenia to provide a definitive answer to the low vulnerability hypothesis.

### Limitations

The findings presented here could be limited by several factors. Due to the nature of the parent study, all participants were required to pass a urine drug screen and not meet current criteria for any drug dependence. This was intended to minimize confounds of current intoxication or withdrawal on measures of cognitive control and performance while in the scanner. While some participants had a history of cannabis abuse or dependence, those who participated in the study either had already stopped using or were able to discontinue for several weeks to participate. This group may ultimately not be representative of individuals with schizophrenia who have ongoing cannabis abuse or dependence. For instance, some studies of first-episode individuals with active cannabis use and/or use disorders actually show lower cognitive performance in users or a lack of difference between current users and nonusers.^75–77^ Another limitation, consistent with many other studies of cannabis use, was the limited information on the composition (ie, percent THC and CBD:THC ratio) of the cannabis used by participants. In addition to cannabis composition, we did not have frequency of use information available for approximately half of the cannabis-using sample although age of initiation was available for the majority of the sample. Correlations with cannabis use metrics were also considered exploratory and not corrected for multiple comparisons. Future neuroimaging studies can benefit from a more comprehensive assessment of cannabis use patterns over time and replication of these relationships.

## Conclusions and Future Directions

The current study found higher cognitive control performance and DLPFC recruitment in FES+CAN compared to FES-CAN. However, an earlier age of starting cannabis use was associated with lower IQ and functioning, and heavier use history was linked to more severe positive symptoms. These findings reinforce the complex relationship between cannabis use, cognition, and psychosis outcomes. One of the primary challenges to understanding these relationships is the retrospective and cross-sectional nature of many existing studies as well as the lack of reliable information on the composition, potency, and frequency of cannabis use. Ongoing prospective studies, such as the Adolescent Brain Cognitive Development study,^78^ may provide some additional insight into the role of premorbid IQ and functioning, as well as genetic and other neurobiological factors, in the association of cannabis use in increasing risk for psychosis as well as paradoxically showing evidence for higher cognition.

## Supplementary Material

sgae016_suppl_Supplementary_Materials
