# Supplementary material for: Using Task-fMRI to Explore the Relationship Between Lifetime Cannabis Use and Cognitive Control in Individuals With First-Episode Schizophrenia
Source: Schizophr Bull Open. 2024 Jul 24;5(1):sgae016. doi: 10.1093/schizbullopen/sgae016 (PMC11317632; doi:10.1093/schizbullopen/sgae016)
Supplement: sgae016_suppl_Supplementary_Materials [file sgae016_suppl_supplementary_materials.docx]

Supplementary Materials

Additional Parent Study Details:

Data analyzed in the present manuscript represent a subset of data collected between 2005 and 2013 as part of a parent study aimed at evaluating cognitive control performance and fronto-parietal brain activity and connectivity in individuals with first episode schizophrenia (NIH: R01MH059883). As part of the larger study design, individuals participated in a diagnostic interview, clinical symptom ratings session, EEG session, and MRI scan. Individuals were primarily recruited through the University of California, Davis Medical Center Early Detection and Preventative Treatment (EDAPT) clinic, which specializes in early psychosis treatment. Clinical interviews were conducted by clinicians with masters or doctoral degrees trained to high reliability (kappa>0.80; range 0.80-1.0). All participants were followed longitudinally and diagnoses were confirmed 12 months after ascertainment.

AX-CPT fMRI Sequence Details:

For the AX-CPT, T2*-weighted echoplanar imaging data were acquired with the following settings: TR=2,000-msec, echo time=40-msec, flip angle=90˚, field of view=22cm. Functional images consisted of 24 contiguous and interleaved 4.0-mm axial slices with a 3.4-mm² in-plane resolution.
